# Development and Clinical Validation of a Novel 5 Gene Signature Based on Fatty Acid Metabolism-Related Genes in Oral Squamous Cell Carcinoma

**DOI:** 10.1155/2022/3285393

**Published:** 2022-11-28

**Authors:** Yi Fan, Jing Wang, Yaping Wang, Yanni Li, Sijie Wang, Yanfeng Weng, Qiujiao Yang, Chen Chen, Lisong Lin, Yu Qiu, Jing Wang, Fa Chen, Baochang He, Fengqiong Liu

**Affiliations:** ^1^Department of Epidemiology and Health Statistics, Fujian Provincial Key Laboratory of Environment Factors and Cancer, School of Public Health, Fujian Medical University, Fuzhou, China; ^2^Key Laboratory of Ministry of Education for Gastrointestinal Cancer, Fujian Key Laboratory of Tumor Microbiology, Fujian Medical University, Fujian, China; ^3^Central Laboratory, Quanzhou First Hospital Affiliated to Fujian Medical University, China; ^4^Department of Oral and Maxillofacial Surgery, The First Affiliated Hospital of Fujian Medical University, Fujian, China; ^5^Laboratory Center, The Major Subject of Environment and Health of Fujian Key Universities, School of Public Health, Fujian Medical University, Fujian, China

## Abstract

**Background/Aim:**

Lipid metabolism disorders play a crucial role in tumor development and progression. The aim of the study focused on constructing a novel prognostic model of oral squamous cell carcinoma (OSCC) patients using fatty acid metabolism-related genes.

**Methods:**

Microarray test and data from The Cancer Genome Atlas (TCGA) were used to identify differentially expressed genes related to fatty acid metabolism. The quantitative real-time polymerase chain reaction (qRT-PCR) was then used to validate the expression of targeted fatty acid metabolism genes. A risk predictive scoring model of fatty acid metabolism-related genes was generated using a multivariate Cox model. The efficacy of this model was assessed by time-dependent receiver operating characteristic curve (ROC).

**Results:**

14 fatty acid metabolism-related genes were identified by microarray test and TCGA database analysis and then confirmed by PCR. Finally, a 5 gene signature (ACACB, FABP3, PDK4, PPARG, and PLIN5) was constructed and a RiskScore was calculated for each patient. Compared to the high RiskScore group, the low RiskScore group had better overall survival (OS) (*p* = 0.02). The RiskScore derived from a 5 gene signature was a prognostic factor (*HR*: 3.73, 95% CI: 1.38, 10.09) for OSCC patients. The predictive classification efficiencies of RiskScore were evaluated and the area under the curve (AUC) values for 1, 3, and 5 years were 0.613, 0.652, and 0.681, respectively. Then we compared the predictive performance of the prognostic model with or without the RiskScore. The 5 gene-derived RiskScore can improve the predictive performance with AUC values of 0.760, 0.803, and 0.830 for 1, 3, and 5 years OS in prognostic model including the RiskScore. While the predicted AUC values of the model without RiskScore for 1, 3, and 5 years OS were 0.699, 0.715, and 0.714, respectively.

**Conclusion:**

We developed a predictive score model using 5 fatty acid metabolism-related genes, which could be a potential prognostic indicator in OSCC.

## 1. Introduction

Oral cancer was one of the common malignancies in Southeast Asia, contributing to 377,713 new cases and 177,757 deaths in 2020 globally [1]. Oral squamous cell carcinoma (OSCC), which accounts for more than 90% of oral cancers, was characterized by a high degree of malignancy and a poor prognosis [2]. Despite advances in treatment, the prognosis for OSCC remains poor, with a 5-year survival rate of only about 50% of those with advanced disease [3]. Herein, it was imperative to explore the mechanism of carcinogenesis and prognostic markers for the prevention and treatment of OSCC.

Lipid metabolism disorders, a well-known feature of malignant tumors, have been crucial for tumor development and progression [4]. Messenger substances formed by lipids could trigger the activation of signaling axes, including phosphoinositide 3-kinases (PI3Ks) and protein kinase C, which could promote carcinogenesis [5, 6]. Fatty acids (FAs) function as a major component of lipids, which form the basic structure of the cell membrane, and played an important role in tumor cell proliferation, invasion, and metastasis [7]. Numerous research have highlighted the potential role of FA metabolism in carcinogenesis, diagnosis, treatment, and prognosis [8, 9]. To date, much attention has been paid to the molecular mechanism and signal transduction pathway of OSCC triggered by FA metabolism. For example, blocking the expression of differentiation cluster 36 (CD36), which correlates with FA uptake, inhibited OSCC metastasis in mice and humans [10, 11]. Uma et al. discovered that downregulation of FA-binding proteins (FABPs) was associated with metastasis of squamous cell carcinoma of the tongue [12]. Considerable evidence suggested that peroxisome proliferator-activated receptors (PPARs), which mediate lipid biosynthesis, are considered a therapeutic target in head and neck cancer [13, 14]. In addition, increased gene and protein expression of the FA family of transport proteins has been found in the tumor microenvironment [15].

In this study, we used public datasets and a microarray assay test to create and validate an OSCC prognostic signature based on FA metabolism genes. In addition, we conducted a thorough analysis of signature genes in order to improve the clinical utility of the markers.

## 2. Materials and Method

### 2.1. Study Participants and Clinical Sample

Tumor and adjacent masses of OSCC patients were obtained between December 2015 and October 2020 from the First Affiliated Hospital of Fujian Medical University in Fujian Province, China. The inclusion criteria of the patient were as follows: (1) cancers of the lip, oral cavities, and parotid corresponded to codes C00 to C07 according to the 10th revision of the International Classification of Diseases (ICD-10); (2) patients who reside in Fujian Province for more than 10 years; and (3) patients with surgical resection and confirmed by pathological examination. Those with other cancers or who had received any preoperative chemotherapy or radiotherapy were not eligible. All surgically resected samples were taken immediately after resection, then frozen in liquid nitrogen and maintained in -80°C cryopreservation until RNA extraction. Finally, 5 pairs of tumor and adjacent masses were submitted to the Arraystar human mRNA microarray test to obtain mRNA expression profiling, and 90 pairs of tumor and adjacent masses were used to confirm target mRNA expression through quantitative real-time polymerase chain reaction (qRT-PCR).

The clinicopathological data of OSCC patients were obtained from the hospital's electronic medical record system. Patients were followed up by telephone interview every 6 months following surgery until January 2021 or until the patient died. Time from the initial diagnosis to death from any cause or last follow-up was defined as overall survival (OS). Censored data included those who were still alive, those who were lost to follow-up, and those who died from other causes.

The study was approved by the Institutional Review Board of Fujian Medical University and conducted following the ethical standards described in the Declaration of Helsinki.

### 2.2. Microarray Assay Test

mRNA expression profiling was obtained from 5 pairs of tumors and matching adjacent mass by microarray test. As described previously [16], CapitalBio Technology Human mRNA Array v4 (4 × 180 K format, Capitalbio Technology Corporation Co., Ltd., Beijing, China) was used for microarray analysis, which included detection probes for 34,235 human mRNAs, as well as 4974 Agilent control probes. The Agilent G2565CA Scanner was used to scan the arrays (Agilent Technologies, Santa Clara, California). Agilent Feature Extraction software was used to evaluate the array images (v10.7). Agilent GeneSpring software was used to perform quantile normalization and further data processing.

### 2.3. TCGA Data Downloading and Preprocessing and GO Analysis

The Cancer Genome Atlas (TCGA) data from patients with head and neck squamous cell carcinoma (HNSCC) were obtained from the official website of UCSC Xena (https://xenabrowser.net/datapages/). mRNA sequencing data of 502 tumor masses and 44 adjacent masses were used to obtain differentially expressed genes (DEGs). The significant DEGs were considered as log2|FC| > 1.0 and the false discovery rate (FDR) < 0.05.

DEGs were submitted to Gene Ontology (GO; http://www.geneontology.org) for functional enrichment analysis to obtain related metabolism pathways.

### 2.4. RNA Extraction and qRT-PCR

Following the manufacturer's recommendations, total RNA was isolated from tumor masses using TRIzol reagent (Invitrogen, Thermo Fisher Scientific, Inc., Waltham, Massachusetts). Using the PrimeScript RTase reagent Kit, 1.0 ug total RNA was reverse transcribed into first-strand cDNA (Takara, Dalian, China). The ABI 7500 System (Applied Biosystems, Carlsbad, California) was used to perform the qRT-PCR with 2.0 ul cDNA using the SYBR PrimeScript RT-PCR kit (Takara, Dalian, China). GAPDH was used as an internal control. The target genes' relative expression levels were calculated using the 2^−ΔΔCt^ method. The primer sequences of these mRNAs were provided in Supplementary Table 1. The described details were shown in Supplement Material and Methods.

### 2.5. Statistical Analysis

R software was used to conduct the statistical analysis (version 4.1.1). Mann–Whitney *U* test was used to compare gene expression levels. Correlation between two variables was evaluated by Pearson or Spearman coefficient. The coefficients of multivariate Cox regression model (*β*) were multiplied by the relative expression levels of DEGs to obtain the prognostic RiskScore.

Kaplan−Meier (KM) curves and the log-rank test were used to compare survival rates. A RiskScore was calculated by multiplying the coefficients (*β* value) of a multivariate Cox regression model in which all 5 genes were included by their corresponding expression level. The predictive performance of RiskScore for OS was evaluated using the time-dependent receiver operating curve (ROC) and decision curve analysis (DCA). Independent prognostic factors were investigated using univariate and multivariate Cox regression. The nomogram was used to visualize the results of multivariate Cox regression analysis, performance of which was evaluated by calibration curves.

All of the tests were two-sided. A result with a *p* value < 0.05 was considered statistically significant.

## 3. Result

### 3.1. Identification of the DEGs Related to FA Metabolism

**Figure 1:**
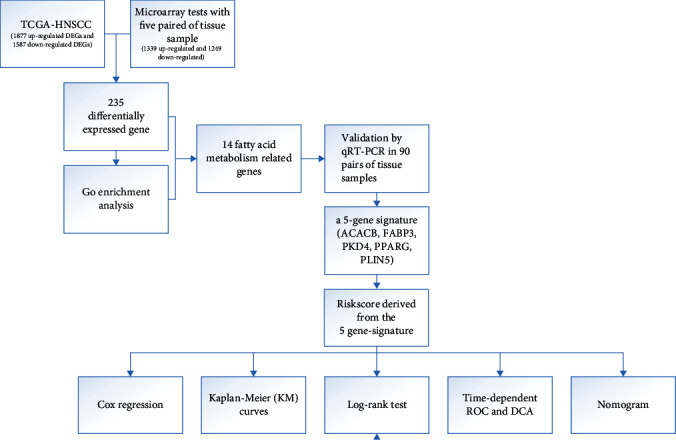
Flowchart of the study. DEGs: differentially expressed genes; TCGA: the cancer genome atlas; HNSCC: head and neck squamous cell carcinoma.

**Figure 2:**
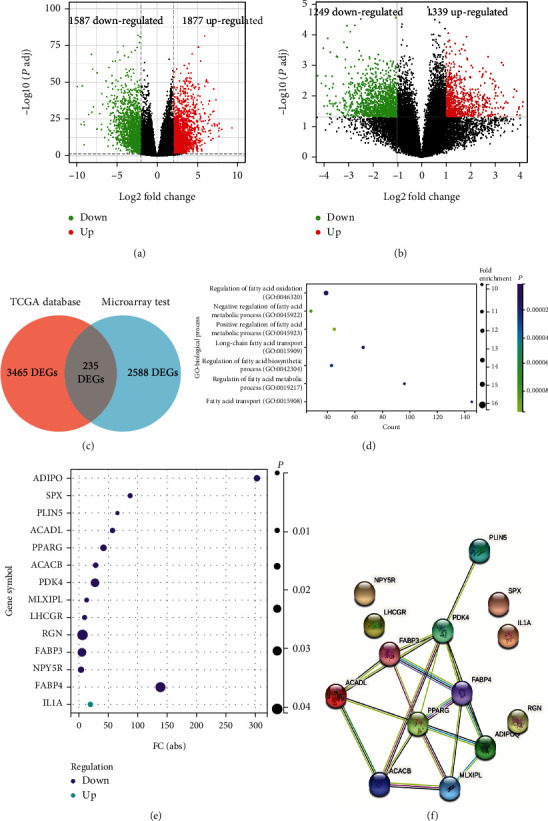
Screening for fatty acid metabolism-related genes by microarray test and TCGA database. (a) Volcano plot of differentially expressed genes of HNSCC in TCGA. (b) Volcano plot of differentially expressed genes by microarray test. (c) Venn plot of DEGs in TCGA-HNSCC and microarray test. (d) Fatty acid metabolism-related pathways identified by GO analysis. (e) 14 fatty acid metabolism-related genes that were commonly identified in the two gene sets. (f) Protein interaction network analysis of fatty acid-related genes.

The study design was presented in a flowchart (Figure 1). mRNA sequencing data of 502 tumor samples and 44 adjacent normal samples of HNSCC patients were downloaded from TCGA and a total of 3465 DEGs were identified of which 1877 genes were upregulated and 1587 genes were downregulated (Figure 2(a)). While 2588 DEGs (1339 upregulated and 1249 downregulated) were identified from microarray test with 5 pairs of tumors and matching adjacent normal samples, results of which were shown in the volcano plot (Figure 2(b)). 235 common DEGs of the two gene sets were identified and presented in the Venn plot (Figure 2(c)). The 235 DEGs were then submitted to GO analysis and found to be enriched in 41 GO annotations (Supplement Table 2), which included 7 FA metabolism-related processes (Figure 2(d)). A total of 219 genes are involved in these 7 FA metabolism-related processes, which were then cross-verified with 235 DEGs. Finally, 14 genes, which were not only differentially expressed but also related to FA metabolism-related processes, were selected. A bubble plot was constructed to visualize their expression profiles (Figure 2(e)). Furthermore, a Protein-Protein Interaction Network (PPI) analysis was performed for these 14 genes and the result was shown in Figure 2(f), demonstrating the hub role of the FABP3, PPARG, ACACB, and PDK4.

### 3.2. Validation of DEGs Related to FA Metabolism by qRT-PCR

mRNA expressions of 14 DEGs were validated by qRT-PCR in 90 pairs of OSCC tumor and adjacent samples. The expression level of 5 DEGs can only be detected in a limited number of tumor masses and thus be excluded. Relative expression levels of the remaining 9 DEGs, including LHCGR, FABP3, NPY5R, PPARG, RGN, PLIN5, ACACB, PDK4, and FABP4, were shown in Supplement Figure 1. The expression levels of FABP3, PPARG, PLIN5, ACACB, and PDK4 in oral cancer samples were significantly lower than those in adjacent normal samples (all *p* < 0.05) and then were included in the prognosis analysis.

### 3.3. Construction of the Prognostic Risk Model

**Figure 3:**
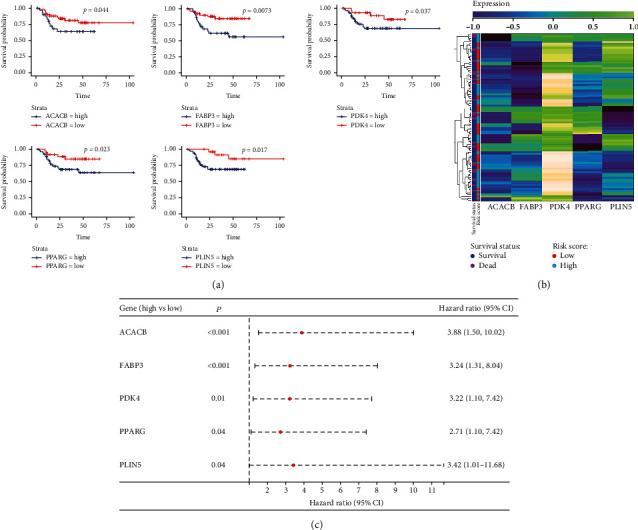
Identification and validation of 5 fatty acid metabolism-related gene signature by qRT-PCR. (a) The Kaplan−Meier survival curve of 5 gene signature. (b) Heatmap of mRNA expression of 5 gene signature with different RiskScore and survivor status. (c) Forest plot of mRNA expression of 5 gene signature obtained by multivariable Cox model adjusted by TNM stage and lymph node metastasis at diagnosis.

KM curves and log-rank test results of ACACB, FABP3, PDK4, PPARG, and PLIN5 were shown in Figure 3(a). KM curves revealed that the expression levels of ACACB, FABP3, PDK4, PPARG, and PLIN5 were related to the survival of patients with OSCC. Patients in the low-expression level group had higher survival rate than those in the high-expression level group of ACACB (*p* = 0.044), FABP3 (*p* = 0.0073), PDK4 (*p* = 0.037), PPARG (*p* = 0.023), and PLIN5 (*p* = 0.017). The global expression changes of ACACB, FABP3, PDK4, PPARG, and PLIN5 in 90 OSCC patients were visualized by a heatmap (Figure 3(b)). In general, expression of ACACB, FABP3, PDK4, PPARG, and PLIN5 was lower in the survival group, which was consistent with the results presented in the KM curve. In addition, several potential prognostic factors of OSCC were explored by Cox regression analysis. TNM stage (HR: 2.85, 95% CI: 1.01-10.30) and lymph node metastasis at diagnosis (HR: 2.89, 95% CI: 1.12-8.52) were found to be independently related to survival of patients with OSCC (Table 1). Expression level of ACACB (HR: 3.88, 95% CI: 1.50-10.02), FABP3 (HR: 3.24, 95% CI: 1.31-8.04), PDK4 (HR: 3.22, 95% CI: 1.21-7.72), PPARG (HR: 2.71, 95% CI: 1.01-7.42), and PLIN5 (HR: 3.42, 95% CI: 1.01-11.68) was significantly associated with OS in OSCC after adjusted for TNM stage and lymph node metastasis (Figure 3(c)).

Nextly, the 5 genes significantly related to OSCC prognosis were used to establish a prognostic RiskScore model by the formula below

(1) $$\begin{array}{c} \text{RiskScore}=-0.170Х{\text{Gene}}_{\text{ACACB}}+0.267Х{\text{Gene}}_{\text{FABP}3}+0.119Х{\text{Gene}}_{\text{PDK}4}+0.180Х{\text{Gene}}_{\text{PPARG}}+0.097Х{\text{Gene}}_{\text{PLIN}5}. \end{array}$$

The coefficients (*β* value) of 5 genes were derived from a multivariate Cox regression model in which all 5 genes were included (Table 2). And the corresponding partial correlation coefficient of the 5 genes with the RiskScore was also calculated and shown in Table 2.

The RiskScore was calculated for each patient based on the expression levels of the 5 genes and was divided into high and low score groups. As shown in Supplement Figure 2, the expression levels of ACACB, FABP3, PDK4, PPARG, and PLIN5 were lower in the low RiskScore group than in the high RiskScore group (all *p* < 0.05), which was consistent with the partial correlation results between RiskScore and the 5 genes.

**Figure 4:**
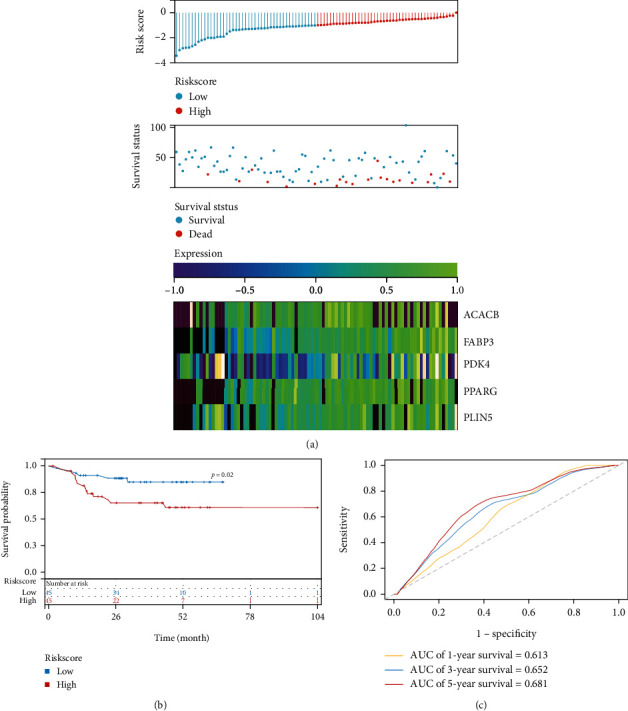
Construction of RiskScore and evaluation of prognostic performance. (a) Distribution of RiskScore and survival status of 5 genes in OSCC patients. (b) The Kaplan−Meier survival curve of RiskScore. (c) Time-dependent ROC of RiskScore in predicting 1 year, 3 year, and 5-year survival status.

And next, the distribution of RiskScore in patients with OSCC was described in Figure 4(a). The proportion of death of patients with high RiskScore was significantly higher than that of patients with low RiskScore, which suggested that patients with high RiskScore had worse prognoses. KM curve was plotted according to low and high RiskScore as shown in Figure 4(b), and a better OS was found in patients with low RiskScore compared with those with high RiskScore (*p* = 0.02). The predictive classification efficiencies of the model were then examined, and the area under the curve (AUC) values for 1, 3, and 5 years OS were 0.613, 0.652, and 0.681, respectively (Figure 4(c)).

### 3.4. Comparison of Different Prognosis Models and Construction of Nomogram

Furthermore, Cox regression analyses were used to investigate the prognostic independence of the RiskScore (Table 1). Results showed that RiskScore (HR: 3.73, 95CI: 1.38-10.09) was independently associated with survival of patients with OSCC after adjusting for age, sex, BMI, tobacco smoking, alcohol drinking, oral hygiene, TNM stage, tumor size, tumor site, and lymph node metastasis at diagnosis. This finding suggested that the RiskScore, which was derived from 5 genes, was an independent prognostic factor.

**Figure 5:**
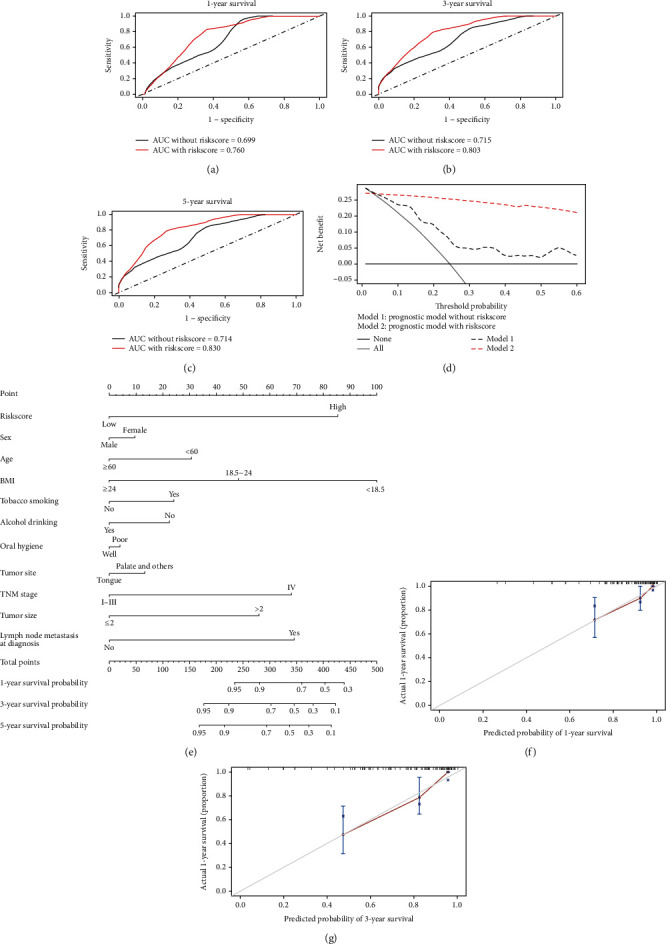
Comparison of different prognostic model and construction of nomogram. (a−c) Comparison of different prognostic models with and without RiskScore for 1-year, 3-year, and 5-year survival by time-dependent ROC. (d) DCA curve of prognostic model with and without RiskScore. (e) Construction of nomogram with RiskScore and clinical. (f, g) Calibration curves of the nomogram.

Then, time-dependent ROC and DCA were used to evaluate the predictive performance of the prognostic model with or without the 5 gene signature-derived RiskScore. The predicted AUC values of the model without RiskScore for 1, 3, and 5 years OS were 0.699, 0.715, and 0.714, respectively. While the predicted AUC values of the model with RiskScore for 1, 3, and 5 years OS were 0.760, 0.803, and 0.830, respectively (Figures 5(a)–5(c)), which indicated improved predictive performance by RiskScore. As shown in DCA curve (Figure 5(d)), a higher net benefit of the model with RiskScore was observed in clinical treatment. Moreover, a nomogram was constructed according to the improved prognostic model to predict 1-year, 3-year, and 5 years OS (Figure 5(e)). And the accuracy of the model and potential model overfit was assessed and shown in the calibration curve (Figures 5(f) and 5(g)), in which the predictions fell on a 45 degree diagonal line.

### 3.5. Correlation Analysis between Clinical Characteristics and 5 Genes

**Figure 6:**
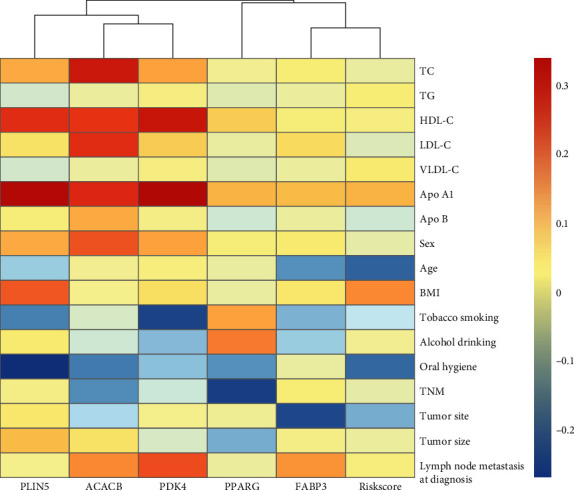
Association between the 5 genes, RiskScore, and clinicopathological features in OSCC patients.

We further visualized the association between the 5 genes, RiskScore, and clinicopathological features in patients with OSCC, and the results were shown in Figure 6 and Supplement Table 3. Age, tobacco smoking, and tumor size were inversely correlated with the RiskScore, while BMI, tumor site, and lymph node metastasis at diagnosis were positively associated with the RiskScore. Moreover, oral hygiene exhibited a general negative correlation with the 5 genes and RiskScore. Interestingly, positive correlations were observed between the 5 genes, RiskScore, and blood lipid indicators (including TC, TG, HDL-C, VLDL-C, Apo A1, and Apo B), which provided supportive evidence that the 5 genes may play important role in the lipid metabolism regulation.

## 4. Discussion

OSCC is a common malignant tumor that can arise at any site in the mouth cavity. Although significant advances have been made in the development of comprehensive treatment strategies for OSCC, effective prognostic biomarkers and therapeutic targets are still lacking. In order to improve patient prognosis and develop potential therapy, it is critical to identify genetic factors that drive tumor progression and contribute to unfavorable outcomes. Recent studies have revealed an expanded range of roles played by lipids in the development and progression of human cancers including oral cancer.

Tumor cells have different metabolic requirements than normal cells, which have been widely reported [17]. FA metabolism as a potential target for cancer treatment has received considerable attention, and targeted inhibition of FA uptake has been an effective strategy for patient survival [18, 19]. Numerous studies, including animal studies and epidemiological studies, have shown that FA metabolism was involved in malignant tumor progression and tumor resistance [20, 21], and several tumor-related FA metabolic pathways have been identified that were correlated with poor prognosis in glioblastoma, squamous cell carcinoma of the lung, and hepatocellular carcinoma [22–24]. Abnormal expression of enzymes involved in de novo lipogenesis has been reported to be a promising target for oncotherapy [25, 26]. However, most of the previous studies have focused on the association between FA metabolism and malignant tumors, which are mainly derived from glandular epithelium [18, 27–29], and few studies have reported the association between FA metabolism and OSCC.

In the present study, we used TCGA database and microarray test to construct a risk predictive scoring model consisting of 5 FA metabolism-related genes. The FA metabolism-related genes included in the risk predictive scoring model have already been reported in human cancers. ACACB served as an inhibitor of FA oxidation and studies showed that inhibition of ACACB reduced cell proliferation in breast carcinoma and hepatocellular carcinoma [30]. Previous studies establishing the importance of de novo lipogenesis in tumor progression and the knockout of ACACB genes in mice indicated a crucial role of ACACB in liver carcinogenesis [31]. In recent clinical studies, FABP3 has been linked to tumor growth, but its functions have been contradictory. Increased FABP3 expression has been reported to be involved in the progression and aggressiveness of gastric cancer [32]. It was also reported that the expression of FABP3 was significantly increased in tumor mass compared to adjacent mass in non-small-cell lung cancer and higher expression of FABP3 was an independent prognostic factor in non-small-cell lung cancer [33]. However, FABP3 has been reported to act as a tumor suppressor in breast cancer, and its transfection into breast cancer exhibited an antiproliferative effect [34]. PDK4, an important regulator of cellular energy metabolism, was found to be relatively highly expressed in several cancers [35]. Regulation of PDK4 was an important regulator of ferroptosis resistance in carcinogenesis and tumor progression [36]. PPARG regulates the peroxisomal *β*-oxidation pathway of FAs and was an important regulator of adipocyte differentiation and glucose homeostasis. Studies suggest that PPARG has been associated with tumor prognosis [37], and maybe a therapeutic target for OSCC [14, 38]. The close correlation of PLIN5 with FA metabolism and its involvement in maintaining lipid homeostasis by inhibiting lipolysis have made it a therapeutic target as well as a prognostic biomarker in tumors [39, 40]. Thus, the RiskScore of FA metabolism-related genes reflected in the weighted sum of 5 genes has shown expected predictive performance in the current study and exhibited potential to become new biomarkers for OSCC.

We further leveraged the complementary value of the FA metabolism-related RiskScore to prognosis of OSCC and found that the inclusion of the RiskScore improved the ability to predict patients with OSCC beyond traditional clinicopathological features. The novel RiskScore of 5 FA metabolism-related genes provided new perspectives for identifying OSCC at high risk of mortality. All cancers are generally acknowledged to share common pathogenesis involving multiple stages and multiple genes [41]. Studies investigating different prognostic models of varied tumors have shown that the inclusion of gene biomarkers may outperform the classical prognostic model and be valuable for tailored treatment [42]. Jin et al. [43] found that the prognostic model associated with ferroptosis-related lncRNA may be better than the traditional model in OSCC and provides a new perspective for OSCC therapy. Lipid metabolism-based prognostic models have been developed for colorectal cancer, glioblastoma, and breast cancer, which proved that a gene-based prognostic model can support clinically individualized treatment [44–46]. Patient outcomes can be greatly improved by customized treatments guided by biomarkers that embodied individual differences in tumor genetic and biological characteristics.

Although this study is based on multiomics data analysis and was clinically validated, it still has several limitations. Firstly, the clinical sample size is small and still needs validation in larger patient cohorts. Secondly, although the 5 genes used to establish the RiskScore have been widely investigated in cancers, we did not conduct in vitro experiments to confirm their roles in OSCC cell lines. The underlying mechanisms remain to be elucidated by further studies. Thirdly, we mainly focused on fatty acids metabolism-related genes in the model development and did not include all the common DEGs and GO annotations identified. The potential of developing a panel of markers with optimized predictive ability irrespective of their function need further validation in future studies.

## 5. Conclusion

In conclusion, we identified a 5 gene signature-derived prognostic RiskScore that was an independent prognostic indicator for OSCC patients. Inclusion of the 5 gene signature exhibited superior predictive performance compared with classical prognostic model in OSCC. This study may provide new perspectives in the development of new biomarkers and therapeutic targets for OSCC.

## Figures and Tables

**Table 1:** Univariate and multivariate Cox regression analyses of potential prognostic factors in patients with OSCC.

Variable	Univariate analysis		Multivariate analysis	
	HR (95% CI)	*P*	HR (95% CI)	*P*
Age (y)				
≤60	Reference		Reference	
>60	0.71 (0.29, 1.70)	0.439	0.63 (0.22, 1.73)	0.823
Sex				
Male	Reference		Reference	
Female	0.91 (0.35, 2.35)	0.851	1.15 (1.32, 4.18)	0.363
BMI				
18.5~	Reference		Reference	
18.5~24	0.34 (0.09, 1.16)	0.086	0.45 (0.09, 2.03)	0.299
24~	0.20 (0.03, 1.20)	0.078	0.21 (0.02, 1.69)	0.144
Tobacco smoking				
No	Reference		Reference	
Yes	1.01 (0.42, 2.38)	0.977	1.45 (0.42, 5.02)	0.557
Alcohol drinking				
No	Reference		Reference	
Yes	0.83 (0.33, 2.05)	0.685	0.71 (0.21, 2.36)	0.573
Oral hygiene				
Well	Reference		Reference	
Poor	1.37 (0.58, 3.22)	0.473	1.06 (0.35, 3.21)	0.913
TNM stage				
I-III	Reference		Reference	
IV	3.26 (1.04, 11.09)	0.045	2.85 (1.01, 10.30)	0.048
Tumor size (cm)				
≤2	Reference		Reference	
>2	0.86 (0.36, 2.05)	0.741	2.36 (0.75, 7.39)	0.138
Tumor site				
Tongue	Reference		Reference	
Others site	2.36 (0.79, 7.01)	0.123	1.23 (0.46, 3.23)	0.680
Lymph node metastasis at diagnosis				
No	Reference		Reference	
Yes	3.84 (1.40, 10.49)	0.009	2.89 (1.12, 8.52)	0.033
RiskScore				
Low	Reference		Reference	
High	2.91 (1.13, 7.52)	0.027	3.73 (1.38, 10.09)	0.009

**Table 2:** The coefficients between the 5 genes and survival of OSCC by multivariate Cox analysis.

Gene symbol	Full name	Coefficient (*β* value)	Partial correlation with RiskScore#
ACACB	Acetyl-CoA carboxylase beta	-0.170	0.256^∗∗^
FABP3	Fatty acid binding protein 3	0.267	0.793^∗∗^
PDK4	Pyruvate dehydrogenase kinase 4	0.119	0.364^∗∗^
PPARG	Peroxisome proliferator activated receptor gamma	0.180	0.612^∗∗^
PLIN5	Perilipin 5	0.097	0.429^∗∗^

^#^adjusted for sex and age. ^∗∗^Correlation was significant at the 0.01 level.

## Data Availability

The data that support the findings of this study are available from the corresponding author upon reasonable request.

## Supplementary Materials

Supplementary Materials Supplement Figure 1: relative expression levels of LHCGR, FABP3, NPY5R, PPARG, RGN, PLIN5, ACACB, PDK4, and FABP4 in 90 pairs of tumor and adjacent masses. (A) LHCGR, (B) FABP3, (C) NPY5R, (D) PPARG, (E) RGN, (F) PLIN5, (G) ACACB, (H) PDK4, and (I) FABP4. Supplement Figure 2: expression level of ACACB, FABP3, PDK4, PPARG, and PLIN5 between low and high RiskScore groups. (A) ACACB, (B) FABP3, (C) PDK4, (D) PPARG, and (E) PLIN5. Supplement Table 1: the primer sequences of selected genes in qRT-PCR. Supplement Table 2: 41 biological process identified by GO enrichment analysis with 235 DEGs. Supplement Table 3: the association between the 5 genes, RiskScore, and clinicopathological features in OSCC patients.
