# Supplementary material for: Development and Clinical Validation of a Novel 5 Gene Signature Based on Fatty Acid Metabolism-Related Genes in Oral Squamous Cell Carcinoma
Source: Oxid Med Cell Longev. 2022 Nov 28;2022:3285393. doi: 10.1155/2022/3285393 (PMC9722305; doi:10.1155/2022/3285393)
Supplement: Supplementary Materials — Supplement Figure 1: relative expression levels of LHCGR, FABP3, NPY5R, PPARG, RGN, PLIN5, ACACB, PDK4, and FABP4 in 90 pairs of tumor and adjacent masses. (A) LHCGR, (B) FABP3, (C) NPY5R, (D) PPARG, (E) RGN, (F) PLIN5, (G) ACACB, (H) PDK4, and (I) FABP4. Supplement Figure 2: expression level of ACACB, FABP3, PDK4, PPARG, and PLIN5 between low and high RiskScore groups. (A) ACACB, (B) FABP3, (C) PDK4, (D) PPARG, and (E) PLIN5. Supplement Table 1: the primer sequences of selected genes in qRT-PCR. Supplement Table 2: 41 biological process identified by GO enrichment analysis with 235 DEGs. Supplement Table 3: the association between the 5 genes, RiskScore, and clinicopathological features in OSCC patients. [file 3285393.f1.docx]

Development and clinical validation of a novel 5 genes signature based on fatty acid metabolism- related genes in oral squamous cell carcinoma

Yi Fan^1,2†^, Jing Wang^1,2†^, Yaping Wang^1,2^, Yanni Li^1,2^ , Sijie Wang^1,2^, Yanfeng Weng^1,2^, Qiujiao Yang^1,2^, Chen chen^1,2^, Lisong Lin^4^, Yu Qiu^4^, Jing Wang^3^, Fa Chen^1,2^, Baochang He^1,2^, Fengqiong Liu^1,2^

**Supplement Table 1** The primer sequences of selected genes in qRT-PCR

| Sequence | Genes | Sequence of primers(5'-3') |
| --- | --- | --- |
| 1 | LHCGR | F:TGAGCAAATTTGGCTGCTGTAAAC |
|  |  | R:AGTGCAATGTGGACAACTTCAAGG |
| 2 | FABP3 | F:TGCGGGAGCTAATTGATGGAA |
|  |  | R:TTCTCATAAGTGCGAGTGCAAACTG |
| 3 | ADIPOQ | F:CCTGGTGAGAAGGGTGAGAAAG |
|  |  | R:TGAATGCTGAGCGGTATACATAGG |
| 4 | NPY5R | F:GCATTGCTGAGCAGCAGGTATTTA |
|  |  | R:TCTTTGTTGGACAATCCACAGCTTA |
| 5 | SPX | F:CTTGGCTCTTTTCCTGGTGTTT |
|  |  | R:GTAGTTGGGGATTTGGGCTTC |
| 6 | PPARG | F:TCAGGTTTGGGCGGATG |
|  |  | R:CAGCGGGAAGGACTTTATGTATG |
| 7 | RGN | F:CCGTGGATGCCTTTGACTATGAC |
|  |  | R:GTAACAGGCCACCCAGAGCTTC |
| 8 | PLIN5 | F:TGCTGCTCAGCCTGCCATAC |
|  |  | R:AGGACCTTTATTCTGGAGGCAAATC |
| 9 | IL1A | F:CTCAATTGTATGTGACTGCCCAAGA |
|  |  | R:TGGATGGGCAACTGATGTGAA |
| 10 | ACACB | F:CCACTGATAGGCCATGTTTAGCAC |
|  |  | R:AGGACCTCGAGCTATGCGAGA |
| 11 | ACADL | F:GGATCTGTACTCCGCAGCTATTGTC |
|  |  | R:AGCTCCAGGCTCTGTCATTGCTA |
| 12 | PDK4 | F:GCCAGCATGCTTTCATTTGTC |
|  |  | R:CAACTGGTCTGCCAACCTTCA |
| 13 | FABP4 | F:GGAAAGTCAAGAGCACCATAACC |
|  |  | R:GCTCTCTCATAAACTCTCGTGGAA |
| Internal reference | GAPDH | F:GCACCGTCAAGGCTGAGAAC |
|  |  | R:TGGTGAAGACGCCAGTGGA |

**Supplement Table 2** 41 biological process identified by Go enrichment analysis with 235 DEGs

| GO-Biological process | Count | Fold Enrichment | *P* |
| --- | --- | --- | --- |
| regulation of fatty acid metabolic process (GO:0019217) | 102 | 1.14 | 5.08E-08 |
| multicellular organismal process (GO:0032501) | 6972 | 77.86 | 7.65E-08 |
| nucleic acid metabolic process (GO:0090304) | 2219 | 24.78 | 2.46E-07 |
| regulation of lipid biosynthetic process (GO:0046890) | 197 | 2.2 | 6.47E-07 |
| long-chain fatty acid transport (GO:0015909) | 61 | 0.68 | 9.18E-07 |
| biological adhesion (GO:0022610) | 951 | 10.62 | 1.37E-06 |
| regulation of blood pressure (GO:0008217) | 191 | 2.13 | 2.84E-06 |
| cell adhesion (GO:0007155) | 945 | 10.55 | 3.61E-06 |
| fatty acid transport (GO:0015908) | 75 | 0.84 | 3.79E-06 |
| regulation of fatty acid oxidation (GO:0046320) | 33 | 0.37 | 4.09E-06 |
| circulatory system process (GO:0003013) | 497 | 5.55 | 5.04E-06 |
| homophilic cell adhesion via plasma membrane adhesion molecules (GO:0007156) | 168 | 1.88 | 5.12E-06 |
| regulation of lipid metabolic process (GO:0019216) | 407 | 4.55 | 5.31E-06 |
| cellular macromolecule metabolic process (GO:0044260) | 5098 | 56.93 | 6.96E-06 |
| macromolecule metabolic process (GO:0043170) | 6280 | 70.13 | 9.30E-06 |
| cell-cell adhesion via plasma-membrane adhesion molecules (GO:0098742) | 257 | 2.87 | 1.03E-05 |
| monocarboxylic acid transport (GO:0015718) | 149 | 1.66 | 1.13E-05 |
| male genitalia development (GO:0030539) | 23 | 0.26 | 1.26E-05 |
| multicellular organism development (GO:0007275) | 4899 | 54.71 | 1.68E-05 |
| regulation of cellular ketone metabolic process (GO:0010565) | 194 | 2.17 | 1.84E-05 |
| anatomical structure development (GO:0048856) | 5294 | 59.12 | 1.92E-05 |
| blood circulation (GO:0008015) | 405 | 4.52 | 1.95E-05 |
| response to insulin (GO:0032868) | 239 | 2.67 | 2.43E-05 |
| cell-cell adhesion (GO:0098609) | 510 | 5.7 | 2.52E-05 |
| drug metabolic process (GO:0017144) | 47 | 0.52 | 2.53E-05 |
| regulation of fatty acid biosynthetic process (GO:0042304) | 47 | 0.52 | 2.53E-05 |
| animal organ development (GO:0048513) | 3244 | 36.23 | 3.66E-05 |
| regulation of sequestering of triglyceride (GO:0010889) | 14 | 0.16 | 3.93E-05 |
| muscle system process (GO:0003012) | 294 | 3.28 | 3.97E-05 |
| system development (GO:0048731) | 4313 | 48.17 | 4.21E-05 |
| tissue development (GO:0009888) | 1762 | 19.68 | 4.54E-05 |
| nitrogen compound metabolic process (GO:0006807) | 7032 | 78.53 | 6.00E-05 |
| developmental process (GO:0032502) | 5757 | 64.29 | 6.07E-05 |
| RNA processing (GO:0006396) | 922 | 10.3 | 6.18E-05 |
| response to peptide hormone (GO:0043434) | 401 | 4.48 | 6.47E-05 |
| positive regulation of small molecule metabolic process (GO:0062013) | 149 | 1.66 | 6.75E-05 |
| negative regulation of fatty acid metabolic process (GO:0045922) | 36 | 0.4 | 8.53E-05 |
| positive regulation of fatty acid metabolic process (GO:0045923) | 37 | 0.41 | 9.59E-05 |
| glucose homeostasis (GO:0042593) | 197 | 2.2 | 1.07E-04 |
| carbohydrate homeostasis (GO:0033500) | 198 | 2.21 | 1.11E-04 |
| nucleobase-containing compound metabolic process (GO:0006139) | 2713 | 30.3 | 1.12E-04 |

**Supplement Table 3** The association between the 5 genes, RiskScore and clinicopathological features in OSCC patients

|  | ACACB | FABP3 | PDK4 | PPARG | PLIN5 | RiskScore |
| --- | --- | --- | --- | --- | --- | --- |
| TC | 0.291** | 0.063 | 0.156 | 0.026 | 0.144 | 0.008 |
| TG | 0.012 | 0.012 | 0.045 | -0.013 | -0.034 | 0.064 |
| HDL-C | 0.256* | 0.056 | 0.298** | 0.111 | 0.263* | 0.044 |
| LDL-C | 0.264* | 0.092 | 0.112 | 0.007 | 0.078 | -0.017 |
| VLDL-C | 0.011 | 0.013 | 0.042 | -0.015 | -0.035** | 0.066 |
| Apo A1 | 0.267* | 0.135 | 0.339** | 0.144 | 0.333 | 0.139 |
| Apo B | 0.145 | 0.010 | 0.038 | -0.042 | 0.055 | -0.039 |
| Age | 0.025 | -0.153 | 0.047 | 0.007 | -0.093 | -0.203 |
| Sex | 0.220* | 0.069 | 0.155 | 0.057 | 0.148 | 0.001 |
| BMI | 0.029 | 0.075 | 0.084 | 0.005 | 0.216* | 0.171 |
| Tobacco smoking | -0.030 | -0.113 | -0.236* | 0.150 | -0.170 | -0.066 |
| Alcohol drinking | -0.041 | -0.090 | -0.108 | 0.182 | 0.069 | 0.027 |
| Oral hygiene | -0.174 | 0.004 | -0.102 | -0.152 | -0.271* | -0.197 |
| TNM stage | -0.158 | 0.064 | -0.048 | -0.243* | 0.038 | -0.001 |
| Tumor size | -0.080 | -0.234* | 0.034 | 0.020 | 0.076 | -0.121 |
| Tumor site | 0.078 | 0.037 | -0.030 | -0.123 | 0.134 | 0.015 |
| Lymph node metastasis at diagnosis | 0.170 | 0.162 | 0.229* | 0.014 | 0.031 | 0.047 |

*Correlation was significant at the 0.05 level

** Correlation was significant at the 0.01 level


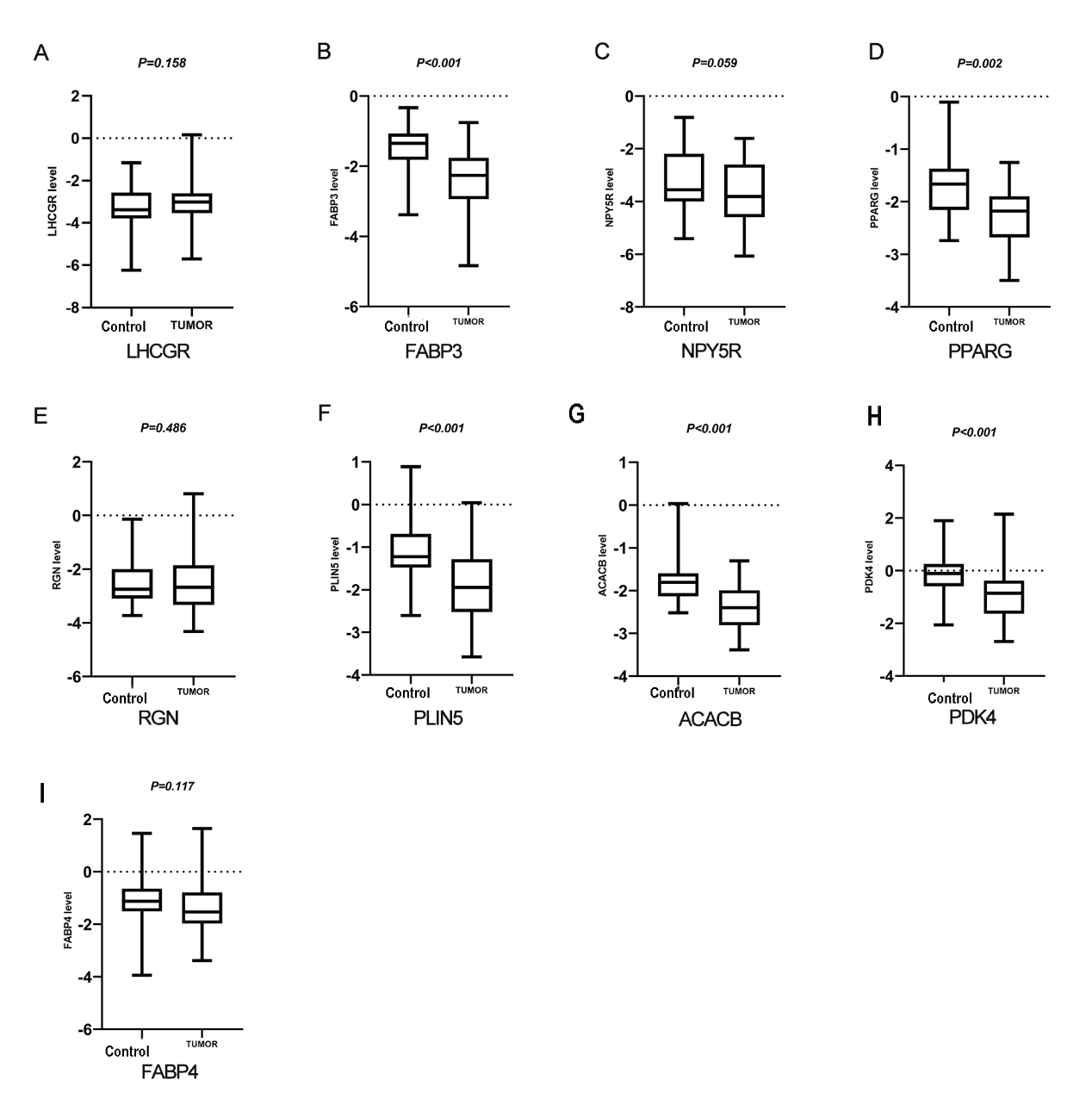


**Supplement Figure 1** Relative expression levels of LHCGR, FABP3, NPY5R, PPARG, RGN, PLIN5, ACACB, PDK4, and FABP4 in 90 pairs of tumor and adjacent masses. (A) LHCGR (B) FABP3 (C) NPY5R (D) PPARG (E) RGN (F) PLIN5 (G) ACACB (H) PDK4 (I) FABP4


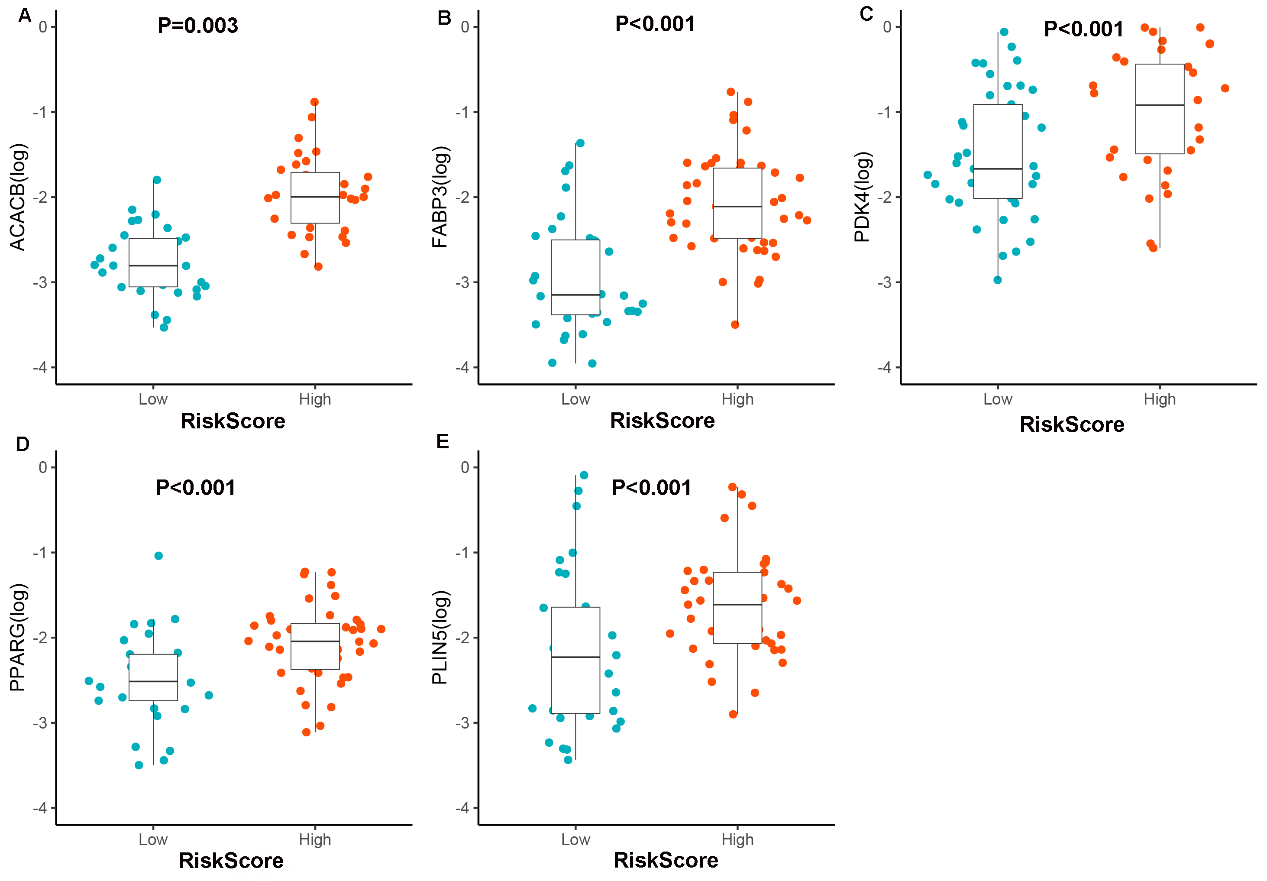
**Supplement Figure 2** Expression level of ACACB, FABP3, PDK4, PPARG, and PLIN5 between low and high RiskScore groups. (A) ACACB (B)FABP3 (C)PDK4 (D)PPARG (E)PLIN5
